# The efficacy of mindfulness-based therapy for anxiety, social skills, and aggressive behaviors in children and young people with Autism Spectrum Disorder: A systematic review

**DOI:** 10.3389/fpsyt.2023.1079471

**Published:** 2023-03-13

**Authors:** Tundi Loftus, Danielle C. Mathersul, Michelle Ooi, Shu H. Yau

**Affiliations:** ^1^School of Psychology, Murdoch University, Murdoch, WA, Australia; ^2^Centre for Molecular Medicine and Innovative Therapeutics, Health Futures Institute, Murdoch University, Murdoch, WA, Australia; ^3^War Related Illness and Injury Study Center (WRIISC), Veterans Affairs Palo Alto Health Care System, Palo Alto, CA, United States; ^4^School of Education, University of Bristol, Bristol, United Kingdom

**Keywords:** autism, anxiety, social skills, aggressive behaviors, yoga, mindfulness, children, young people

## Abstract

**Introduction:**

The purpose of this systematic review was to examine the efficacy of mindfulness-based interventions for improving anxiety, social skills, and aggressive behaviors in children and young people (CYP) with Autism Spectrum Disorder (ASD); summarize the results across clinic, home, and school contexts; and evaluate the quality of these interventions for clinical practice.

**Methods:**

A search of the PsycINFO, Medline (Ovid), Web of Science, and Scopus databases was conducted in June 2021, and no date restrictions were applied. Inclusion criteria were quantitative or qualitative research implementing a mindfulness-based intervention for CYP aged 6–25 years with a diagnosis of ASD, Pervasive Development Disorder, or Asperger’s Syndrome.

**Results:**

We identified 23 articles for inclusion including within subject pre- and post-testing, multiple baselines, and randomized control trials, among other research designs. Of these, a quality analysis conducted using an ASD research-specific risk of bias tool found over half (14) were of weak methodological quality, whereas only four and five were found to be of strong and adequate quality, respectively.

**Discussion:**

While the results of this systematic review suggest promising evidence for the use of mindfulness-based interventions to improve anxiety, social skills, and aggressive behaviors in CYP with ASD, results should be interpreted with caution due to the limitations resulting from the overall weak quality of the studies.

The review protocol was pre-registered on PROSPERO (CRD42021259125) and can be viewed at https://www.crd.york.ac.uk/prospero/display_record.php?RecordID=259125

## Introduction

Autism Spectrum Disorder (ASD)^1^ is a neurodevelopmental disorder characterized by difficulties in social interaction and communication, along with restricted and repetitive behaviors (1, 2). While prevalence rates vary across studies, it is estimated approximately one in 44 Americans have a diagnosis of ASD (3). A 2018 survey conducted by the Australian Bureau of Statistics (ABS) estimated there were ~205,200 individuals with ASD in Australia (4). This is a considerable increase from the estimated 164,000 individuals with ASD in 2015 (4) and may result from improved awareness of the symptoms and presentation of ASD (5), along with broadening criteria for diagnosis in the *Diagnostic and Statistical Manual of Mental Disorders* [5th ed.; DSM–5; (2)].

Early intervention for children and young people (CYP) with ASD demonstrates efficacy in improving cognitive, adaptive, and social–communicative outcomes (6) and may prevent challenges such as anxiety from worsening with age (7).

### Anxiety in CYP with ASD

Mental health symptomology is more common in CYP with ASD compared to the general population (8–10). In the United States, nearly 78% of children with ASD have a comorbid mental health condition [Kerns et al. (11)]; anxiety is one of the most common of these, with ~50% of individuals with ASD experiencing clinically significant symptoms of anxiety that impact on daily life (12). Further, it is estimated that 84% of CYP with ASD experience sub-clinical symptoms of anxiety (10), which typically warrant intervention and are predictors of increasing severity (13).

There are several possible explanations for the high rates of anxiety observed among CYP with ASD. First is the interaction between deficits related to ASD, such as social communication difficulties and environmental factors (e.g., parenting and peer relationships), resulting in increased vulnerability to anxiety (14). Second is emotional regulation (ER) which is described as a person’s ability to effectively manage and respond to an emotional experience (15). Third, the cognitive mechanism of Intolerance of Uncertainty (IU), characterized by the inability to cope with unfamiliar or uncertain situations (16), is commonly observed among CYP with ASD. The presence of ER and IU offer an explanation for anxiety in the younger ASD population, as a multitude of changes and unpredictability occur during childhood and adolescent years which are often beyond their coping abilities (10, 17).

### Social skills in ASD

Childhood and adolescence are a crucial time for the development and acquisition of social skills and behavioral regulation (18). These skills – including social communication, reciprocity, engagement, willingness to interact with others, and ability to form meaningful relationships – are particularly challenging for CYP with ASD (2). This contributes to feelings of social isolation and loneliness, both of which have been identified as common experiences of CYP with ASD (19), who often struggle with the unpredictability of everyday social situations, resulting in maladaptive behavioral responses to social interactions (20). As such, adapting to the changes experienced throughout development, such as the transition from childhood to adolescence, can be especially challenging for CYP with ASD (21). Yet, while CYP with ASD struggle to develop and maintain interpersonal relationships, their desire for these relationships is similar to the general population (22).

### Aggressive behaviors in ASD

CYP with ASD often have difficulty effectively regulating or expressing their emotions (23) which manifests in aggressive behaviors (24). Among individuals with developmental disorders, aggressive behaviors are one of the strongest predictors of the need for crisis intervention, admission to residential care facilities, and the prescription of psychotropic medications (24). These behaviors become particularly challenging as children age and the potential severity of risk of harm to self and others increases.

### Mindfulness-based interventions

Mindfulness – the practice of maintaining awareness of the present moment, while being conscious and attentive to this awareness, in a non-judgemental way (25) – is moderately effective in improving symptoms of anxiety and depression (26) and reducing emotional dysregulation by increasing emotional awareness and acceptance (27) for adults without ASD. Mindfulness-based interventions also improve stress, anxiety, depression, and cognitive performance among CYP without ASD (28, 29). Recently, a meta-analysis of mindfulness-based interventions for individuals with ASD and their caregivers found significant improvements in subjective wellbeing for all participants (30). Yet, only four systematic reviews exist of mindfulness-based interventions for CYP with ASD (31–34) and all have limitations that we aim to address in the current systematic review, as outlined below (35).

First, a systematic review conducted by Cachia et al. (32) examined the efficacy of mindfulness-based interventions on psychological, social, and behavioral outcomes in CYP and adults with ASD. This review included six studies published prior to February 2016 (32). Findings suggested the interventions were efficacious in improving a range of psychological, social, and behavioral problems for individuals with ASD (32). Only six children and 29 adolescents were included in the total 161 participants in the review conducted by Cachia et al. (32). As most of the participants included were adults (*N* = 126), only two of the interventions reviewed by Cachia et al.(32)were tailored to the specific needs of CYP. Further, this review had restrictive definitions of mindfulness-based interventions. Specifically, authors omitted “yoga” and “mind–body therapy,” which are holistic interventions that include mindfulness. These movement-based mindfulness interventions may be more efficacious for CYP with ASD due to a lack of dependence on cognitive ability, a limitation for this population in other interventions, such as Cognitive Behavior Therapy (35).

Second, Hourston and Atchley (33) conducted an exploratory systematic review to investigate the types of mind–body therapies used for individuals with ASD and the outcomes which are targeted (33). Consistent with findings reported by Cachia et al. (32), of the 16 studies included in the review, improvements in mental health were reported in multiple studies (33). While many of the studies reported a decrease in aggressive behaviors, resulting from a lack of controlled trials and lengthy follow-up periods, authors were unable to draw any conclusions on the efficacy of the interventions for behavioral outcomes (33). There were no criteria for the outcomes included in the systematic review, and as such, there was considerable variance in the outcomes of the studies. Further, participants of all ages were included, meaning intervention studies targeting adults with ASD and the parents and caregivers of CYP with ASD were also included in this review.

Third, unlike the reviews conducted by Cachia et al. (32) and Hourston and Atchley (33), Semple (34) focused specifically on interventions targeting CYP with ASD. Semple (34) conducted a systematic review of the effects of mindfulness-based interventions on core symptoms of ASD. Social skills and aggressive behaviors are captured within diagnostic criteria for ASD, meaning these outcomes were included in the review. Improvements were observed across all eight studies included in the review, providing promising evidence that mindfulness-based interventions may be efficacious in improving prosocial behaviors among CYP with ASD (34). The impacts of mindfulness-based interventions upon psychological wellbeing, particularly levels of anxiety, were not considered in this review (34).

Last, (31) conducted a systematic review assessing the effect of Yoga for musculoskeletal function, cardiovascular function, neurological function, and behaviors of CYP with ASD. Only two of the 18 studies in this review included outcome measures of communication and social interaction. While positive results were found in both studies, authors state there is insufficient research with relevant outcome measures to determine the effects of Yoga for improving behaviors of CYP with ASD (31).

### The current review

In summary, only three systematic reviews of mindfulness-based interventions for CYP with ASD exist. None captures the effects of mindfulness-based interventions on anxiety, social behaviors, and aggressive behaviors in CYP with ASD. Additional limitations exist surrounding the sample population age range and/or the definition of mindfulness-based intervention. All authors emphasized the limited and often poor-quality research with individuals with ASD and the importance of further research into mindfulness-based interventions for this population (31–34). Furthermore, since the publication of these reviews, mindfulness-based interventions have grown in popularity and are more commonly used in psychotherapy (36). Together, this highlights the need for an updated review.

The purpose of the current systematic review is to answer the question: Does mindfulness-based therapy – including yoga, mind–body, and mindful-movement based interventions – improve anxiety, social behaviors, and aggressive behaviors in behavior CYP aged 6–25 years with ASD? Doing so will provide a novel understanding of the efficacy of mindfulness-based interventions specifically for CYP with ASD, focusing upon outcome measures related to anxiety, social skills, and aggressive behaviors. A risk of bias analysis will inform whether the methodological quality of the research has improved since the reviews conducted by Cachia et al. (32), Hourston and Atchley (33), Artchoudane (31), and Semple (34). Further, the review will summarize the results across clinic, home, and school contexts, and evaluate the quality of the studies for clinical practice. Assessing these differences provides an opportunity to draw general conclusions about the efficacy of mindfulness-based interventions for CYP with ASD across a range of different settings.

## Methods

The articles were assessed by two independent researchers, who each determined whether the study was suitable for inclusion. All research articles published prior to the June 2021 search date were included in the review, with no date restrictions applied. Due to the research team’s limited translation abilities, articles published in languages other than English were not included in the review. The review protocol was pre-registered on PROSPERO (CRD42021259125).

### Participants

For the purpose of the current review, “young people” are defined as those aged between 6 and 25 years based upon the Australian Department of Health definition of “young adults” (*Support for Young Adults (Ages 18–25)*, n.d.). Participants under the age of six were not included in the review as children younger than this are unlikely to participate effectively in a mindfulness-based intervention. In addition, research on mindfulness interventions for children commonly focuses on ages six and above [Weare (37)]. CYP with a diagnosis of ASD, Pervasive Developmental Disorder (PDD) or Asperger Syndrome were included, in accordance with the DSM-IV and DSM-5 diagnostic criteria (2, 38) and/or ICD-10 (39).

### Intervention

Mindfulness-based interventions in this review included both mindfulness-only interventions as well as mindful-movement/mind–body therapies such as yoga that contain significant mindfulness components. Interventions targeting the parent or caregiver were not included.,

### Control

Studies were not required to have a control or comparison group in order to be included in the review.

### Outcomes

The three outcomes of interest were anxiety, social skills, and aggressive behaviors. Studies were included if they had a measure of anxiety, social skills, such as increased social responsiveness, or aggressive behaviors, such as physical outbursts. Outcomes can be measured through interviews and a range of standardized questionnaires, including the Social Responsiveness Scale [SRS; (40)] and Child Behavior Checklist [CBCL; (41)], among other measures. Studies with an outcome not relevant to anxiety, social skills or aggressive behaviors were excluded.

### Study design

Both qualitative and quantitative research which implemented a mindfulness-based intervention across any context (i.e., home, clinic, school) were included in the review. Relevant reviews and meta-analyses were consulted as sources; however, they were not included in the analysis. Grey literature, including non-peer reviewed articles and unpublished studies, were excluded from the current review as this data is more likely to be inaccurate or incomplete [Benzies et al. (42)].

#### Search and selection strategy

The following electronic databases were searched on 22 June 2021: PsycINFO, Medline (OVID), Web of Science and Scopus. The search string was structured based on the four strings of: Diagnosis, Intervention, Outcome and Population. Specific search strings were adapted for each database. These strings were developed with the assistance of an experienced librarian and the joint author and senior author of the study. The search strings for each database are available in Appendix A.

#### Risk of bias analysis

The quality of the research was independently assessed by two researchers using the Reichow et al. (43) and Reichow (44) risk of bias tool. This has been adapted from Cochrane’s collaboration tool (45) and is specific to evaluating quality of studies in autism research. The tool assesses the methodology and potential biases that may impact the findings (43, 44). An overall rating of a study’s methodological quality, either strong, adequate, or weak, is then awarded (43, 44).

## Results

### Study selection

**Figure 1:**
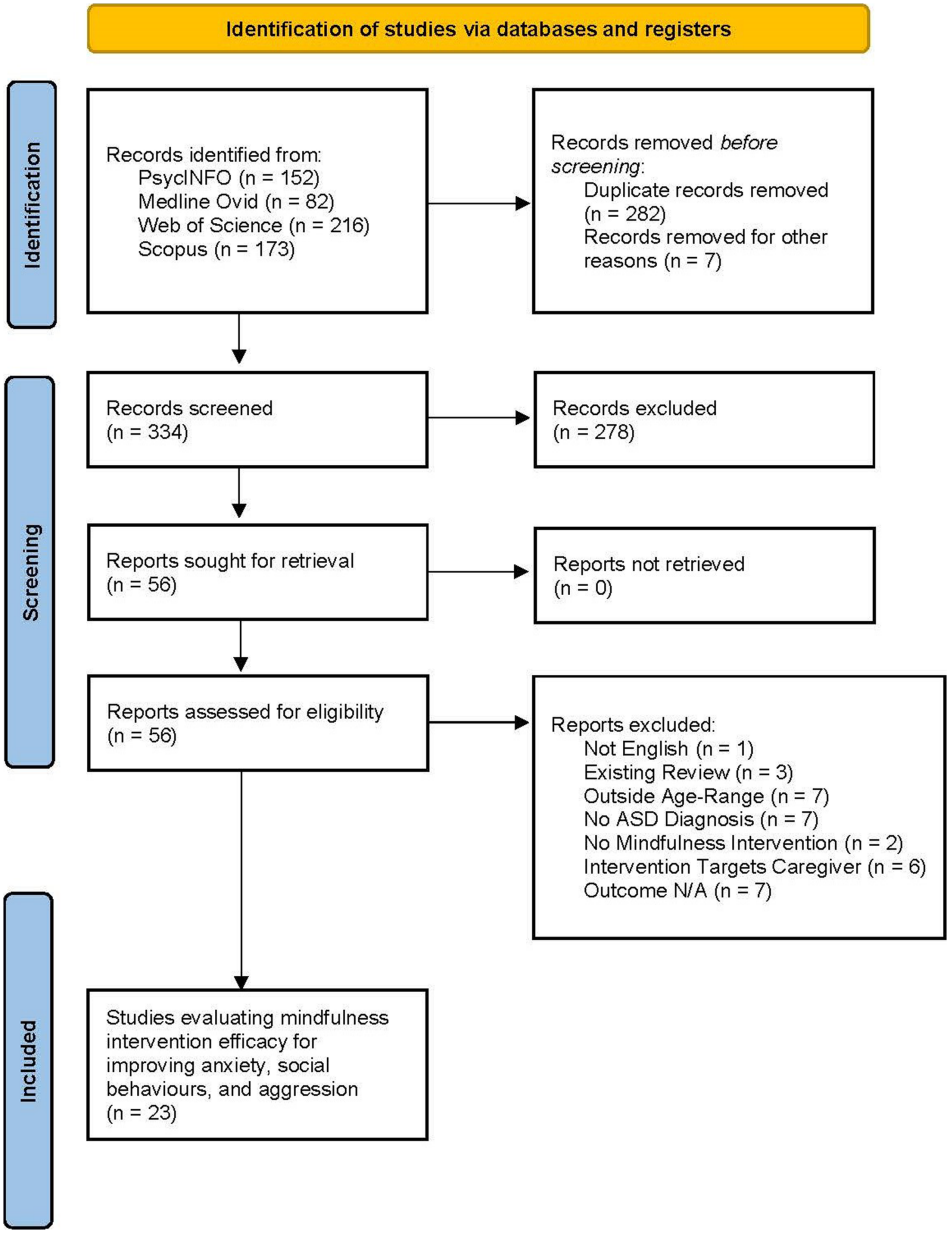
Article selection process – PRISMA table drawn from Page et al. (68).

A total of 334 articles were screened and 56 full-text articles were retrieved for further review. Of these, 23 articles were included in the larger systematic review, to evaluate the efficacy of mindfulness-based interventions for improving psychological well-being, anxiety, social skills, and aggressive behaviors in CYP with ASD. All 23 studies were included in the current review, examining the effects on anxiety, social skills, and aggressive behaviors. The articles were peer reviewed and published between 2010 and 2021. The study selection and screening process is available to review in the PRISMA flow chart (Figure 1).

### Study characteristics

#### Participants

There was a total of 436 participants, ranging between 1 and 61 (*M* = 18.96) participants in each study. The age of participants was reported by 18 studies, with the mean age being 14.8 years. 21 of the 23 articles, totalling 386 participants, reported on participant gender, 81.25% of which were male. The remaining 2 articles (46, 47) did not report participant characteristics beyond diagnosis, therefore the sex of 50 participants is unknown. Further information on the demographics of participants can be viewed in Table 1.

**Table 1:** Characteristics of studies included in systematic review.

Citation	Design	Participants				Intervention type	Relevant outcome measures	Country, context and instructor(s)	Relevant findings and effect sizes
		*Demographics*			*Control/Comparison*				
		Total *n* (% male)	*M* age years (SD); Range years	Diagnosis (measures)					
Ahemaitijiang et al. (48)	Multiple-baseline	*n* = 3 (100%)	15.3 (1.2); 14–17	ASD (DSM V)	–	Soles of the feet (SoF)	Parent Report	China, home – mothers	Aggressive behaviors reduced *p* < 0.05 Tau-U = −0.96
Black and Rosenthal (49)	Case studies	*n* = 6 (83.3%)	18.8 (3.4); 16–24	Autistic Disorder (DSM-IV-TR)	–	Transcendental meditation (TM)	Clinical interviews: participants	Home – self-instructed	Improvements in anxiety, social skills, and aggressive behaviors
Chan et al. (50)	Randomized control trial	*n* = 40 (90%)	12.4 (3.3); 6–17	Autistic disorder (17), PDD (3) (DSM-IV-TR)	PMR *N* = 20 ASD	Nei Yang Gong Chinese Meditation	Parent report, ATEC	China, Clinic – Clinical Psychologists	Social skills improved *p* < 0.05 Cohen’s *d* = 0.68 and aggressive behaviors reduced *p* < 0.05 Cohen’s *d* = 0.66
Conner et al. (51)	Within subject pre- and post-comparison	*n* = 17 (88.2)	14.9 (1.54); 12–17	ASD (ADOS-2)	–	Emotion awareness and skills enhancement program (EASE)	ABC-I, PROMIS anxiety and depression scales	America, Clinic – Therapists	Aggressive behaviors reduced *p* < 0.05 Cohen’s *d* = 0.76, anxiety improved (parent report) *p* < 0.05 Cohen’s *d* = 0.59, no improvement in self report anxiety
de Bruin et al. (67)	Within subject pre- and post-comparison	*n* = 23 (73.9%)	15.8 (2.7); 11–23	Autistic Disorder (4), Asperger’s (8), PDD (11), (DSM-IV-TR, ADOS)	–	MYmind	SRS PSWQ	Clinic – Mental Health Professionals	Social skills improved, *p* < 0.05 Cohen’s *d* = 0.14 to 0.40, no improvement in anxiety Cohen’s *d* => 0.40
Hartmann et al. (52)	Single subject	*n* = 7 (57.1%)	21 (2.6); 18–24	ASD	–	DBT mindfulness	SRS-2, BPAQ, SPAI-23	Clinic – Clinicians and Assistants	Social skills improved, *p* < 0.05 and no reduction in aggressive behaviors or improvement in anxiety
Ho et al. (53)	Randomized control trial	*n* = 37 (76%)	13 (2.3); 10–18	ASD (DSM V)	Waitlist *N* = 18, ASD	MYmind	SRS, CBCL	China, Clinic – Psychologists and Social Workers	Improvement in social skills (treatment and control) Cohen’s *d* => 0.40 and aggressive behaviors
Hwang et al. (54)	Case study	*n* = 6 (83.3%)	11.5 (2.2); 8–15	ASD (3), Asperger’s (2), PDD (1)	–	Mindfulness training	CBCL	Home – Mothers	Aggressive behaviors reduced, *p* < 0.05
Joshi and Rathi (46)	Randomized control trial	*n* = 30 –	– 4–16	ASD (ISAA)	Control *N* = 15 ASD	Yoga	ABC, SCQ	India, Clinic – Therapist and Teachers	No significant improvement in social skills or aggressive behaviors
Kemeny et al. (55)	Randomized crossover	*n* = 27 (74%)	16.3 (2.77); 12–21	ASD	Therapeutic riding	HeartMath (HM)	SRS-2	Clinic – HeartMath Specialist	No improvement in social skills
Pahnke et al. (56)	Quasi-experimental two-group	*n* = 28 (75%)	– 13–21	Autistic disorder (DSM-IV)	Waitlist *N* = 13 (92%) ASD	ACT mindfulness	SDQ, Self- and Educator Report	Sweden, School – Psychologists and Teacher	Social skills improved, *p* < 0.05 ɲp^2^ = 0.06
Radhakrishna (57)	Within subject pre- and post- comparison	*n* = 6 (83.3%)	– 8–14	Autistic disorder (DSM-IV-TR)	–	Yoga	Clinical Interviews: Parent and Educator	Clinic – Yoga Instructor	Improvement in social skills
Ridderinkhof et al. (17)	Within subject pre- and post-comparison	*n* = 45 (80%)	13 (2.72); 8–19	Autistic disorder (DSM-IV and ADOS-G)	–	MYmind	SRS, CBCL, YSR	Netherlands, Clinic – Mental Health Professionals	Social skills improved *p* < 0.05 Cohen’s *d* = >0.40, and aggressive behaviors reduced *p* < 0.05 Cohen’s *d* => 0.40
Ridderinkhof et al. (58)	Qualitative	*n* = 14 (57%)	12.4 (2.4); 9–17	ASD (DSM-5 and ADOS)	–	MYmind	Self- and Parent Report	Netherlands, Clinic – Mental Health Professionals	Improvement in social skills
Salem-Guirgis et al. (59)	Within subject pre- and post-comparison	*n* = 23 (82.6%)	15.6 (2.6); 12–23	ASD	–	MYmind	SRS-2	Canada, Clinic – Psychologists and Behavior Therapists	Social skills improved *p* < 0.05 Cohen’s *d* => 0.40
Scroggins et al. (60)	Case study	*n* = 1 (100%)	7 –	Apert Syndrome and ASD	–	Yoga	STBC, TRIAD	Clinic – Yoga Instructor	Improvement in social skills
Sharma and Sharma (47)	Between subjects	*n* = 20 –	– 8–14	ASD	Control	Yoga	Parent and educator report	India, School	Aggressive behaviors reduced *p* < 0.05
Singh et al. (61)	Multiple-Baseline	*n* = 3 (100%)	15.3 (2.05) 13–18	Asperger’s	–	Meditation on the Soles of the Feet (SoF)	Self-, Parent and Sibling Report	Home – Mothers	Aggressive behaviors reduced
Singh et al. (62)	Multiple-Baseline	*n* = 3 (100%)	15.6 (1.24) 14–17	ASD	–	Meditation on the Soles of the Feet (SoF)	Parent and sibling report	Home – Mothers	Aggressive behaviors reduced
Singh et al. (63)	Multiple-Baseline	*n* = 4 (100%)	11.2 (0.82) 10–12	ASD (DSM V)	–	SOBER Breathing Space	Parent report	Clinic – Mindfulness Trainer	Aggressive behaviors reduced
Singh et al. (64)	Multiple-Baseline	*n* = 3 (100%)	16.6 (0.47) 16–17	ASD	–	Surfing the Urge	Parent and Educator Report	Clinic – Mindfulness Trainer	Aggressive behaviors reduced, *p* < 0.05
Sotoodeh et al. (65)	Randomized control trial	*n* = 29 (72.4%)	11.2 (2.91) 7–15	ASD (DSM V and ADI-R)	Control *n* = 14 (72.4%)	Yoga	ATEC-II	Iran, Clinic – Therapists	Social skills improved, *p* < 0.05
Tanksale et al. (66)	Randomized Control Trial with mixed repeated measures	*n* = 61 (67.7%)	9.4 (1.3); 8–12	ASD	Waitlist control *n* = 30 (60% male), ASD	Incredible Explorers group program combining Yoga-informed Mind Body Practice with Cognitive Behavioral Therapy	ASC EAQ BRIEF-2	Australia, Clinic – Clinical psychologist and psychology students	Reduced performance anxiety *p* < 0.05 Cohen’s *d* => 0.40, no improvement in other sub-types of anxiety

ABC and -I, Aberrant Behavior Checklist and Irritability Subscale; ASC, Anxiety Scale for Children; ATEC and -II, Autism Treatment Evaluation Checklist and Second Edition; BPAQ, Buss Perry Aggression Questionnaire; BRIEF-2, Behavior Rating Inventory of Executive Function; CBCL, Child Behavior Checklist; EAQ, Emotional Awareness Questionnaire; PSWQ, Penn State Worry Questionnaire; PROMIS, Patient Reported Outcomes Measurement Information System; SCQ, Social Communication Questionnaire; SDQ, Social Difficulties Questionnaire; SRS and SRS-2, Social Responsiveness Scale and 2nd Edition; SPAI-23, Social Phobia and Anxiety Inventory; STBC, Sparks Target Behavior Checklist; TRIAD, Treatment and Research Institute for ASD Social Skills Assessment; YSR, Youth Self Report (CBCL).

Of the 436 participants, 407 had a diagnosis of ASD, 15 had a diagnosis of Pervasive Developmental Disorder (PDD), 13 had a diagnosis of Asperger’s Syndrome, and one had a diagnosis of Apert Syndrome and ASD. Ten of the research articles excluded participants who had a comorbid diagnosis, intellectual disability, or an IQ level below 80 (17, 46, 50–53, 58, 59, 65, 66). An additional three of the articles included only participants requiring low levels of support (48, 56, 57). Therefore, participants who required higher levels of support, and/or were minimally verbal, were excluded from these studies.

#### Movement- or mind-based mindfulness interventions

There was substantial variance in the types of mindfulness-based interventions used in the studies included in this review. The majority (13 out of the 23) studies used movement-based mindfulness-based interventions. Six out of these 13 studies used a yoga-based intervention (46, 47, 57, 60, 65, 66); and five studies used the Mymind program (17, 53, 58, 59, 67). The final two movement-based interventions were Nei Yang Cong Chinese meditation (50) and Mindfulness training (54).

Nine of the studies used non-movement mindfulness-based interventions. Most of these were mindfulness-only interventions: three used Soles of the Feet (SoF) mindfulness training in (48, 61, 62), one each used HeartMath (55), SOBER (Stop, Observe physical and emotional sensations, Breathe, Expand awareness, and Respond) Breathing Space (63), and Surfing the Urge (64). The remaining three studies used mindfulness-based CBT: the Emotion Awareness and Skills Enhancement (EASE) program (51), Dialectical Behavior Therapy (52), and Acceptance and Commitment Therapy (56). Black and Rosenthal (49) did not provide details of the procedure used for Transtheoretical Mindfulness; therefore, it is unknown whether this mindfulness-based intervention incorporated movement or psychological therapy.

#### Intervention modifications for CYP with ASD

Twelve of the studies included in the review made modifications to the intervention protocols utilized to suit specific needs of CYP with ASD (17, 51–56, 58, 59, 65–67). Modifications typically included the use of simplified language, visual prompts, and focus upon relevant situations such as social interactions. Most notably, de Bruin et al. (67) adapted the MYMind intervention for CYP with ASD. Adaptations to the MYMind protocol included an additional session for repetition, focus on using mindfulness in ASD relevant situations, use of concrete (rather than abstract) language, and clearing detailing each session prior to commencement for predictability. This modified protocol was used in several of the research articles (17, 53, 58, 59).

#### Duration of intervention

The duration of the interventions varied considerably between the studies, from 2 weeks (64) to 1.7 years (47). The most common lengths of the interventions were: five which ran for 9 weeks (17, 53, 58, 59, 67), four lasting 4 weeks (50, 60, 61, 63), and three which ran for 12 weeks (46, 49, 52). The intervention duration for the remaining nine studies can be viewed in Table 1, Appendix B.

#### Intervention instructors

Of the 23 studies, 16 of the research projects were conducted in a clinical setting; 11 of these were implemented by a mental health professional (50, 51), de Bruin et al., 2014; (17, 52, 53, 58, 59, 63–66), three were delivered by yoga instructors (46, 57, 60), and one was facilitated by a HeartMath specialist (55). Five of the programs were implemented in the home: four were conducted by the mothers of the participants (48, 54, 61, 62), and one was a self-administered intervention (49). The remaining two interventions were conducted in the school: one was implemented by the educator (56), and one did not report the instructor of the program (47).

#### Study design

Of the 23 articles reviewed, five used a within subject pre- and post-comparison design (17, 51, 57, 59, 67) and another five used a multiple-baseline design (48, 61–64). Five studies used a randomized control trial, comparing the experiment group to either waitlist control, treatment as usual, or a control group (46, 50, 53, 65, 66). There were two case studies (54, 60) and two qualitative studies, using semi-structured clinical interviews (49, 58). Of the remaining four studies there was one single subject design (52), one randomized crossover (55), one between-subjects design (47), and one quasi-experimental two group trial (56).

#### Outcome measures

The outcome measures relevant to anxiety, social skills, and aggressive behaviors in each study are presented in Table 1. Of the 23 studies, only two used semi-structured clinical interviews to determine intervention effects (49, 58). The remaining studies exclusively used outcome measures based upon observer and/or self-report. There was no consistent outcome measure for anxiety, with all five studies using different measures. The Social Responsiveness Scale (SRS) (40) was used in five of the studies measuring social skills outcomes (17, 52, 53, 55, 67). The Child Behavior Checklist (CBCL) (41) was the most common outcome measure for aggressive behaviors and was used in three of the studies (17, 53, 54). The outcome measures used to evaluate anxiety, social skills, and aggressive behaviors, along with the characteristics of the studies, can be viewed in Table 1. An expanded version of this table, containing additional information, is available in Appendix B.

### Effects of mindfulness on anxiety

Five studies had specific outcomes relating to anxiety, with varying results in improvement following the intervention. There was one qualitative report on a reduction in anxiety post-intervention (49). Conner et al. (51) found a significant reduction in anxiety in parent-reported measures, but no significant changes in self-reports of anxiety. Tanksale et al. (66) found a significant reduction in self-reported performance anxiety, however there were no significant reductions in other sub-types of anxiety. Two studies (52, 67) report no improvements in anxiety symptoms post-intervention. Two of the five studies measuring anxiety included a follow-up analysis ranging from 6 to 9-weeks post intervention (66, 67). The post-intervention improvements found by Tanksale et al. (66) were retained at the time of follow-up.

For outcomes relating to anxiety symptoms an effect size of 0.59, indicating a medium effect size (68), was reported by Conner et al. (51). De Bruin et al. (67) and Tanksale et al. (66) reported effect sizes on the relevant outcome measures to be <0.40. Hartmann et al. (52) did not report effect sizes for anxiety outcomes. Due to the qualitative design of the Black and Rosenthal (49) study, effect sizes were not applicable.

#### Setting

Of the five studies that measured anxiety outcomes, four were conducted in a clinic (51, 52, 66, 67). The results were mixed; one study found a significant reduction in anxiety in parent-reported measures, however not in self-report measures (51), and one study showed trends in the direction of improvement, however only found a significant reduction in self-reported performance anxiety (66). The remaining two clinic-based studies reported no improvements in anxiety symptoms post-intervention (52, 67). One study was conducted in the home, and participants reported lower levels on anxiety (49).

### Effects of mindfulness on social skills

Out of 14 studies, half found an improvement in social skills (17, 50, 52, 56, 59, 65, 67). Scroggins et al. (60) found improvements in social skills; however, the significance values were not reported as the research was a single subject case study. Black and Rosenthal (49) and Ridderinkhof et al. (58) conducted qualitative research, with improvements in social skills observed in both studies. Black and Rosenthal (49) interviewed young people who had undergone a transcendental meditation intervention. Three out of the six participants reported improved social skills (49). Of the remaining four studies focussing on social skills, two found no significant improvement in the treatment group (46, 55). The third found comparable improvement in social skills, measured using the SRS-2, in both the treatment and control group (52). Radhakrishna (57) also found an improvement in social skills, however not at a significant level. Five of the studies measuring social skills included a follow-up analysis, ranging from one to 4 months post-intervention (17, 52, 56, 59, 67). The improvements in social skills observed at the conclusion of the intervention were retained, or increased, at the time of follow-up.

For social skills outcomes Cohen’s *d* effect sizes of 0.68 and 0.40 respectively, indicating a medium to large effect size (69), for the relevant outcome measures were reported by Chan et al. (50) and de Bruin et al. (67). Pahnke et al. (56) expressed effect sizes as partial eta-square with 0.01 reflecting a small effect, 0.06 moderate, and 0.14 a large effect size (69). Pahnke et al. (56) reported a moderate effect size for improvements in social skills (ɲ*p*^2^ = 0.06). Ho et al. (53), Ridderinkhof et al. (17) and Salem-Guirgis et al. (59) reported effect sizes on the relevant outcome measures to be <0.40. Effect sizes were not applicable to three of the studies as two were qualitative studies (49, 58) and one was a single subject case study with no standardized outcome measures (60). The remaining 11 studies did not report the effect sizes for social skills.

#### Setting

Of the 14 clinic-based interventions which examined social skills, 50% reported a significant improvement in social skills (50), de Bruin et al., 2014, (17, 52, 59, 65), 33.3% found trends in the direction of improvement (53, 57, 58, 60), and the remaining 16.7% found no improvement in relevant outcomes (46, 55). A significant improvement in social skills was reported by Pahnke et al. (56) in their school-based intervention. Black and Rosenthal (49) implemented a home-based intervention which found improvements in social skills.

### Effects of mindfulness on aggressive behaviors

Outcomes measuring aggressive behaviors were examined in 14 of the 23 studies in this review (17, 46–54, 61–64). Of these, seven studies reported a significant decrease in aggressive behaviors following a mindfulness-based intervention (17, 47, 48, 50, 51, 54, 64). In Black and Rosenthal’s (49) qualitative study, two of the six participants reported a reduction in behaviors of aggression. Singh et al. (61–63) also found a reduction in aggressive behaviors, however the significance level was not reported in the results of these studies. Four studies measuring aggressive behaviors conducted a follow-up analysis ranging from one to 4 years post-intervention (48, 61–63). All four studies found that the effect of the intervention, a reduction in aggressive behaviors, had been retained or improved at follow-up.

Effect sizes >0.40 for outcome measures related to aggressive behaviors were found for the following two studies: 0.66 (50), and 0.76 (51). Ahemaitijiang et al. (48) reported effect size (−0.96) using the Tau-U statistic, where 0.80 or above indicates a very large effect (70). Ridderinkhof et al. (17) reported effect sizes <0.40 on relevant outcome measures. Effect sizes were not applicable to Black and Rosenthal (49) as this was a qualitative study. The remaining nine articles did not report effect sizes for aggressive behaviors.

#### Setting

Of the eight clinic-based interventions which examined aggressive behaviors, 50% found a significant result in relevant outcome measures (17, 50, 51, 64), 25% found trends in the direction of improvement (53, 63) and 25% found no improvement (46, 52). Out of the five home-based programs, 40% found a significant result in outcome measures of aggressive behaviors (48, 54). The remaining 60% of home-based interventions found a trend toward improvement in aggressive behaviors (49, 61, 62). Sharma and Sharma (47) conducted their study in a school setting and found a significant improvement in aggressive behaviors.

### Quality of studies

The risk of bias tool was used to determine the methodological quality of the studies included in this review. An overall rating of a study’s methodological quality, either strong, adequate, or weak, was then awarded to each study (43, 44). The results of the quality analysis are further detailed below.

#### Primary indicators for group research

The primary quality indicators for group research include six categories: participant characteristics, independent variable, comparison condition, dependent variable, link between research question and data analysis and use of statistical tests (43, 44). The possible ratings for each category are “not applicable”, “unacceptable quality,” “acceptable quality,” and “high quality.” The primary quality indicators ratings given for group research articles are presented in Table 2.

**Table 2:** Primary quality indicators of group research.

**Citation**	**Participant characteristics**	**Independent variable**	**Comparison condition**	**Dependent variable**	**Link between research question and data analysis**	**Use of statistical tests**
Ahemaitijiang et al. (48)	✓✓	✓✓	✗	✓	✓✓	✓
Black and Rosenthal (49)	✓✓	N/A	✗	✓	N/A	N/A
Chan et al. (50)	✓✓	✓✓	✓✓	✓✓	✓✓	✓✓
Conner et al. (51)	✓✓	✓✓	✗	✓✓	✓✓	✓✓
de Bruin et al. (67)	✓✓	✓✓	✗	✓✓	✓✓	✓✓
Hartmann et al. (52)	✓✓	✓	✗	✓✓	✓✓	✓
Ho et al. (53)	✓✓	✓✓	✓✓	✓✓	✓✓	✓✓
Hwang et al. (54)	✓	✓✓	✗	✓✓	✓✓	✓
Joshi and Rathi (46)	✗	✓	✓	✓	✓	✓
Kemeny et al. (55)	✓	✓✓	✓✓	✓✓	✓✓	✓✓
Pahnke et al. (56)	✓✓	✓✓	✓✓	✓✓	✓✓	✓✓
Radhakrishna (57)	✓✓	✓✓	✗	✗	✗	✗
Ridderinkhof et al. (17)	✓✓	✓✓	✗	✓✓	✓✓	✓✓
Ridderinkhof et al. (58)	✓✓	✓✓	✗	✓	✓✓	N/A
Salem-Guirgis et al. (59)	✓	✓	✗	✓✓	✓✓	✓✓
Sharma and Sharma (47)	✗	✓	✓	✗	✓	✓
Singh et al. (61)	✓	✓✓	✗	✓	✓✓	✓
Singh et al. (62)	✓	✓✓	✗	✓	✓✓	✓
Singh et al. (63)	✓✓	✓✓	✗	✓	✓✓	✓
Singh et al. (64)	✓	✓✓	✗	✓	✓✓	✓
Sotoodeh et al. (65)	✓✓	✓✓	✓✓	✓✓	✓✓	✓✓
Tanksale et al. (66)	✓✓	✓✓	✓✓	✓✓	✓✓	✓✓

N/A, not applicable; ✗, unacceptable quality, ✓, acceptable quality, ✓✓, high quality.

#### Secondary indicators for group research

The secondary quality indicators for group research include eight categories: random assignment, interobserver agreement, blind raters, fidelity, attrition, generalization and/or maintenance, effect size and social validity (43, 44). The possible ratings for each category are “not applicable,” “no evidence” and “evidence.”

The secondary quality indicators ratings given for group research articles are presented in Table 3.

**Table 3:** Secondary quality indicators of group research.

**Citation**	**Random assignment**	**Interobserver agreement**	**Blind raters**	**Fidelity**	**Attrition**	**Generalization and/or maintenance**	**Effect size**	**Social validity**
Ahemaitijiang et al. (48)	✗	✓	N/A	✓	✓	✓	✓	✓
Black and Rosenthal (49)	✗	✗	N/A	✗	✓	✗	N/A	✓
Chan et al. (50)	✓	N/A	✓	✗	✓	✗	✓	✓
Conner et al. (51)	✗	N/A	N/A	✓	✓	✓	✓	✓
de Bruin et al. (67)	✗	N/A	N/A	✓	✓	✓	✓	✓
Hartmann et al. (52)	✗	N/A	N/A	✗	✓	✓	✗	✓
Ho et al. (53)	✓	N/A	✓	✓	✓	✗	✗	✓
Hwang et al. (54)	✗	N/A	N/A	✓	✓	✗	✗	✓
Joshi and Rathi (46)	✓	N/A	✗	✗	✓	✗	✗	✓
Kemeny et al. (55)	✓	N/A	✗	✗	✓	✗	✗	✓
Pahnke et al. (56)	✓	N/A	✗	✗	✓	✓	✓	✓
Radhakrishna (57)	✗	N/A	N/A	✗	✓	✗	✗	✓
Ridderinkhof et al. (17)	✗	N/A	✗	N/A	✓	✓	✗	✓
Ridderinkhof et al. (58)	✗	✗	✗	N/A	✓	✗	N/A	✓
Salem-Guirgis et al. (59)	✗	N/A	N/A	✓	✓	✓	✗	✓
Sharma and Sharma (47)	✗	N/A	✗	✗	✓	✗	✗	✗
Singh et al. (61)	✗	✓	✗	✗	✓	✓	✗	✓
Singh et al. (62)	✗	✓	✗	✗	✓	✓	✗	✓
Singh et al. (63)	✗	✓	✗	✓	✓	✓	✗	✓
Singh et al. (64)	✗	✓	✗	✗	✓	✗	✗	✓
Sotoodeh et al. (65)	✗	N/A	✗	✗	✓	✗	✗	✓
Tanksale et al. (66)	✓	N/A	✗	✗	✓	✓	✓	✓

N/A, not applicable; ✗, no evidence; ✓, evidence.

#### Primary and secondary quality indicators for single-subject research

The primary quality indicators for single-subject research are participant characteristics, independent variable, dependent variable, baseline condition, visual analysis, and experimental control. The secondary quality indicators for single-subject research are interobserver agreement, kappa, fidelity, blind raters, generalization and/or maintenance, and social validity. The ratings given for both primary and secondary quality indicators are available to view in Tables 4, 5.

**Table 4:** Primary quality indicators of single-subject research.

**Citation**	**Participant characteristics**	**Independent variable**	**Dependent variable**	**Baseline condition**	**Visual analysis**	**Experimental control**
Scroggins et al. (60)	✓	✓	✓✓	✗	✗	✓

N/A, not applicable; ✗, unacceptable quality; ✓, acceptable quality, ✓✓, high quality.

**Table 5:** Secondary quality indicators of single-subject research.

**Citation**	**Interobserver agreement**	**Kappa**	**Fidelity**	**Blind raters**	**Generalization and/or maintenance**	**Social validity**
Scroggins et al. (60)	N/A	✗	✗	✗	✗	✓

N/A, not applicable; ✗, no evidence, ✓, evidence.

### Strength ratings for included studies

The overall methodological quality of a research article is assessed by the criteria outlined by Reichow et al. (43) and Reichow (44), including both the primary and secondary quality indicators. As reflected in Table 6, of the 23 articles included in the current review, four were found to be of strong methodological quality, five to be adequate quality, and 14 to be weak quality.

**Table 6:** Strength ratings of the research articles.

**Citation**	**Strength**
Ahemaitijiang et al. (48)	Weak
Black and Rosenthal (49)	Weak
Chan et al. (50)	Strong
Conner et al. (51)	Adequate
de Bruin et al. (67)	Adequate
Hartmann et al. (52)	Weak
Ho et al. (53)	Strong
Hwang et al. (54)	Weak
Joshi and Rathi (46)	Weak
Kemeny et al. (55)	Adequate
Pahnke et al. (56)	Strong
Radhakrishna (57)	Weak
Ridderinkhof et al. (17)	Adequate
Ridderinkhof et al. (58)	Weak
Salem-Guirgis et al. (59)	Weak
Scroggins et al. (60)	Weak
Sharma and Sharma (47)	Weak
Singh et al. (61)	Weak
Singh et al. (62)	Weak
Singh et al. (63)	Weak
Singh et al. (64)	Weak
Sotoodeh et al. (65)	Adequate
Tanksale et al. (66)	Strong

## Discussion

The aim of this systematic review was to examine the efficacy of mindfulness-based interventions for improving anxiety, social skills, and aggressive behaviors in CYP with ASD; summarize the results across contexts; and evaluate the quality of the studies for clinical research and practice. A total of 23 studies were included with majority of the findings demonstrating positive intervention effects. Three of the five studies measuring anxiety found an improvement in anxiety following the mindfulness-based interventions, however there was some variance between the parent-reported and self-reported outcomes. Eleven of the 14 studies that measured social skills found improvements across a range of domains such as social responsiveness and willingness to engage with others. All 14 studies measuring aggressive behaviors reported either a significant reduction or a trend toward improvement in aggressive behaviors following the mindfulness-based intervention.

Mindfulness-based interventions may improve anxiety, social skills, and aggressive behaviors by teaching children be aware of their emotions and remain present in the current moment. By increasing awareness and acceptance of emotions through mindfulness-based interventions, individuals have increased capability to cope with novel or unexpected situations, thereby lowering their IU. This awareness and acceptance of emotions also facilitates improved emotional regulation by providing CYP with mindfulness techniques which allow them to cope better with challenging emotions and situations.

A risk of bias analysis determined the overall quality (60%) of the included studies to be weak due to a lack of randomized control trials and comparison conditions, poor intervention fidelity, minimal reporting of effect sizes and a paucity of follow-up data collection. However, the risk of bias analysis found all studies have high social validity and low attrition, with the exception of Sharma and Sharma (47) which did not meet the criteria for social validity. This means the participants, their parents, caregivers and/or educators, reported high satisfaction with the intervention and believed the outcomes to have social importance. This is evidenced by the low attrition rate across all studies, with most participants remaining in the studies once they commenced the intervention.

Among all Australian individuals with ASD in 2018, 68.9% reported to have a profound or severe limitation in at least one of the following areas: communication, self-care, or mobility (4). Almost half (44.1%) of individuals with ASD had profound or severe communication difficulties, meaning they required assistance to communicate with others (4) and remain nonspeaking despite intervention (71). This subgroup of CYP with ASD often have increased difficulty with emotional regulation due to difficulties with effectively communicating their feelings and basic needs with others (72). Modifications were made to the protocols utilized in several of the interventions to make them more suitable for CYP with ASD. This included the use of visual prompts and concrete language to enhance understanding. However, despite these adaptations, many of the studies excluded those who require high levels of support, have a comorbid intellectual disability, and/or IQ below 80. This is likely a result of the perceived difficulty of facilitating an intervention with CYP who require higher levels of support. Excluding a large number of individuals with ASD who have lower functioning ability results in a homogenous sample which does not accurately reflect the spectrum within the ASD community and excludes those who could stand to benefit most.

There is substantial variance in the frequency and duration of the mindfulness-based interventions that have been included in this review. In comparing the two most common intervention durations (9 and 4 weeks), similar results were observed. All studies of these durations reported improvements across outcome measures. However, there were three studies in the 9-week long interventions that reported significant results, compared to only one study reporting significant results in the 4-week long interventions. While this may suggest a longer intervention duration is more efficacious, further research is necessary to determine the impact intervention frequency and duration has upon outcomes.

As discussed, 12 of the studies taught mindfulness from a movement-based perspective, nine studies used a mind-based approach, and one study did not provide detail on the intervention procedure. Both approaches were found to be efficacious, with no notable difference between the outcomes of the movement-based versus mind-based interventions. Many of the studies excluded CYP who require higher levels of support, such as assistance with communication. Therefore, it is not within the scope of this review to determine whether a movement-based intervention, such as yoga, which allows individuals to use physical movements to bring awareness to the present moment (73), may be more efficacious for CYP with ASD who require substantial support with communication.

Only 47.8% of the studies included a follow-up analysis, with data being collected from a range of 1 month–4 years post-intervention. Of these, follow-up analyses found outcomes of the intervention to be maintained or improved after one to 4 months for social skills and one to 4 years for aggressive behaviors. Based on the findings of the research including a follow-up stage, preliminary evidence suggests mindfulness-based interventions provide individuals with ASD with a lasting toolset for handling their emotions in social situations and regulating their emotionally reactive behavior.

Many of the studies included in the review relied upon self- and/or observer-report of the social and aggressive behaviors of participants. Self-report outcome measures alone are not sufficient in evaluating the efficacy of interventions due to the possible influence of social desirability bias and lack of introspective ability among participants (74). Additionally, intervention outcomes have been found to vary depending on whether the measure was self-, clinician-, or observer-report (75). While these measures provide valuable insight into perceived efficacy, we cannot reliably conclude whether the interventions were efficacious based on these measures alone.

Effect sizes represent the practical significance of the results found in a study and can be used to compare different studies (76). It is important in intervention research that effect sizes are reported so practitioners can understand if the intervention has real-world significance and how these results compare to other intervention strategies. While 14 of the included studies received a “no evidence” rating for effect size in the secondary quality indicators table (43, 44), this is largely due to lack of reporting. As previously discussed, of these 14 studies, only four reported a small effect size. As the remaining 10 studies did not report effect size, it remains unknown whether the effects of the interventions would be large enough to be clinically significant for participants.

### Implications

#### Theoretical implications

As previously discussed, it is likely IU has an impact on the social skills of CYP with ASD due to the inherent uncertainty and unpredictability of social interactions. This may lead to loneliness and/or frustration and then aggressive behaviors, among CYP who are not able to communicate effectively and express their emotions. The mindfulness-based interventions used in the studies included in this systematic review provide CYP with ASD strategies to manage their thoughts and emotions by teaching them to pause, be present in the current moment, and observe their feelings before responding. By employing these strategies in unpredictable situations individuals may better be able to control their emotional responses to uncertainty (77). Meaning, these mindfulness-based interventions may enhance tolerance of uncertainty, resulting in a reduction in anxiety and associated internalizing and externalizing behaviors.

The variation in intervention efficacy for anxiety outcomes may be explained by the high reliance on observer and self-report measures. CYP with ASD find it harder to recognize mental health symptomology, as they often have more difficulty with emotional insight (78). Anxiety is a more complex construct to measure than overt social skills and aggressive behaviors. This offers an explanation as to why the outcomes of the mindfulness-based intervention for anxiety were more varied when compared to the overall positive outcomes for social skills and aggressive behaviors.

The findings of this systematic review suggest a variety of mindfulness-based interventions may be efficacious in supporting the improvement of anxiety, social skills, and aggressive behaviors among CYP with ASD. Further, when follow-up analysis has been conducted, results of the intervention are maintained or increased. This provides promising evidence that, when provided with intervention, CYP with ASD can reduce anxiety and enhance their social and emotional regulation skills, with enduring effect. These findings support ongoing research in and the development of mindfulness programs as a complementary intervention targeting anxiety in this population.

#### Clinical practice

There was no notable variance in the efficacy of mindfulness-based interventions when implemented in a clinic or home context. While only two of the studies were conducted in a school setting, both also reported significant results. In addition, interventions instructed by a health professional or parent also reported similar efficacy. This suggests mindfulness-based interventions can be implemented by a range of instructors, in a variety of settings, and be equally successful. This is particularly important due to the limited and often expensive resources targeting anxiety, social skills, and aggressive behaviors for CYP with ASD. Many families experience difficulties in accessing support for CYP with ASD due to the expense associated with ongoing interventions facilitated by clinicians and therapists (79). The findings of the current review suggest trained parents can instruct effective mindfulness-based interventions in the home. Similarly, these interventions could be incorporated by educators within the classroom. This means both parents and educators may make meaningful improvements for children with ASD, at a reduced expense and while on a waitlist for a space in an intervention program.

#### Research recommendations

It is necessary to include a broader range of CYP with ASD in future research to represent the ASD community more accurately. For example, inclusion of those who require higher levels of support or have an IQ below 80, using a co-design method with their families. Despite some use of technology (such as displaying visual prompts on an iPad), the interventions were delivered in-person, which may add additional complications for CYP who require higher levels of support. An online mindfulness-based intervention may enhance accessibility for these individuals. Additionally, the studies included in this review disproportionality focus on male participants. While ASD is three and a half times more likely to occur in males (4), it is important to include more females in future research to deepen our understanding of how mindfulness-based interventions may benefit both sexes.

Due to the poor quality of many of the research articles included in this review, it is important for future research to improve methodological quality. This could be achieved by conducting randomized control trails with blind raters, ensuring intervention fidelity, reporting effect sizes, and conducting follow-up analysis to determine maintenance of the intervention effects. The use of structured clinical interviews, along with standardized outcome measures, would offer a greater breadth of information for future research. This would help to inform how the interventions improve the constructs and is especially beneficial when measuring complex symptomology, such as anxiety. Finally, the development of a manualised mindfulness-based intervention co-designed specifically to address common issues experienced by the target population, including anxiety, social skill deficits, and aggressive behaviors, would enhance intervention fidelity and accessibility.

### Limitations

This review included studies which incorporated mindfulness-based CBT interventions like Acceptance Commitment Therapy (ACT), Dialectical Behavior Therapy (DBT), and Emotion Awareness and Skills Enhancement Program (EASE), however, their use obscures which intervention component(s) led to significant effects. There was also substantial variance in the mindfulness techniques employed in the studies, precluding recommendations for a specific type of mindfulness for CYP with ASD and making it difficult to ascertain which aspects of the mindfulness-based interventions were most successful.

## Conclusion

The present systematic review of PsycINFO, Medline (Ovid), Web of Science and Scopus resulted in 23 research articles on the effects of mindfulness-based interventions for anxiety, social skills, and aggressive behaviors of CYP with ASD. The risk of bias analysis conducted in this review found the methodological quality of the studies to be largely weak due to a lack of randomized control trials and comparison conditions, poor intervention fidelity, minimal reporting of effect sizes and a paucity of follow-up data collection. However, the review found promising evidence in support of the efficacy of mindfulness-based interventions for improving social skills and aggressive behaviors among CYP with ASD. It also found that the interventions can be implemented by parents and educators who have been trained in mindfulness, with similar efficacy to interventions facilitated by clinicians and therapists. As a result of these positive findings, further experimental research addressing the identified methodological issues is recommended.

## Data availability statement

The original contributions presented in the study are included in the article/Supplementary material, further inquiries can be directed to the corresponding author.

## Author contributions

TL, DM, MO, and SY conceived and designed the structure of the review. TL and MO conducted the systematic review, contributed to writing the review, and prepared the tables and figures. The review was edited by DM and SY. All authors contributed to the article and approved the submitted version.

## Conflict of interest

The authors declare that the research was conducted in the absence of any commercial or financial relationships that could be construed as a potential conflict of interest.

## Publisher’s note

All claims expressed in this article are solely those of the authors and do not necessarily represent those of their affiliated organizations, or those of the publisher, the editors and the reviewers. Any product that may be evaluated in this article, or claim that may be made by its manufacturer, is not guaranteed or endorsed by the publisher.

## Footnotes

^1^There is no universally accepted term used to refer to the ASD population, however keeping in line with American Psychological Association recommendations, person-first language (i.e., child with ASD) will be used for the purposes of this review American Psychological Association, D (1). *Disability*. https://apastyle.apa.org/style-grammar-guidelines/bias-free-language/disability#:~:text=In%20person%2Dfirst%20language%2C%20the,or%20%E2809Can%20epileptic%E2%80%9D.
