# Supplementary material for: The efficacy of mindfulness-based therapy for anxiety, social skills, and aggressive behaviors in children and young people with Autism Spectrum Disorder: A systematic review
Source: Front Psychiatry. 2023 Mar 13;14:1079471. doi: 10.3389/fpsyt.2023.1079471 (PMC10040538; doi:10.3389/fpsyt.2023.1079471)
Supplement: Supplementary file 1 [file Data_Sheet_1.docx]

# Appendix A: Search Strings

**PsycINFO**

(autism OR asd OR autistic OR Asperger* OR ("neurodevelopmental disorder" OR "neurodevelopmental disorders") OR "pervasive developmental disorder*") **AND** (mindfulness OR "mindfulness-based therap*" OR "mindfulness-based intervention*" OR yoga OR "mind-body therap*" OR meditation) **AND** (("social behavior" OR "social behaviors") OR "emotional regulation" OR “emotional dysregulation” OR well-being OR wellness OR "mental health" OR anxiety OR depression OR aggress* OR microaggression* OR attention OR stress OR "quality of life" OR communication OR health) **AND** (child* OR youth OR ("young people" OR "young peoples") OR ("emerging adult" OR "emerging adulthood" OR "emerging adults") OR adolescent* OR ("young adult" OR "young adulthood" OR "young adults"))

**Medline (OVID)**

1. ((autism or asd or asperger* or autistic or ("neurodevelopmental disorder" or "neurodevelopmental disorders") or "pervasive developmental disorder*") **and** (mindfulness or "mindfulness-based therap*" or "mindfulness-based intervention*" or yoga or "mind-body therap*" or meditation) **and** ("social behavior" or "social behaviors" or "emotional regulation" or "emotional dysregulation" or well-being or wellness or "mental health" or anxiety or depression or aggress* or microaggression* or attention or stress or "quality of life" or communication or health) **and** (child* or youth or ("young people" or "young peoples") or ("emerging adult" or "emerging adulthood" or "emerging adults") or adolescent* or ("young adult" or "young adulthood" or "young adults"))).mp. [mp=title, abstract, original title, name of substance word, subject heading word, floating sub-heading word, keyword heading word, organism supplementary concept word, protocol supplementary concept word, rare disease supplementary concept word, unique identifier, synonyms]
2. Limit 1 to English language

**Web of Science**

**TOPIC:** ((autism  OR asd  OR autistic  OR Asperger*  OR ("neurodevelopmental disorder"  OR "neurodevelopmental disorders")  OR "pervasive developmental disorder*") **AND** (mindfulness  OR "mindfulness-based therap*"  OR "mindfulness-based intervention*"  OR yoga  OR "mind-body therap*"  OR meditation) **AND** (("social behavior"  OR "social behaviors")  OR "emotional regulation"  OR “emotional dysregulation”  OR well-being  OR wellness  OR "mental health"  OR anxiety  OR depression  OR aggress*  OR microaggression*  OR attention  OR stress  OR "quality of life"  OR communication  OR health)  AND (child*  OR youth  OR ("young people"  OR "young peoples")  OR ("emerging adult"  OR "emerging adulthood"  OR "emerging adults")  OR adolescent*  OR ("young adult"  OR "young adulthood"  OR "young adults")))

**Refined by:** **LANGUAGES:** (ENGLISH )

**Scopus**

( TITLE-ABS-KEY ( autism  OR  asd  OR autistic OR asperger* OR "neurodevelopmental disorder"  OR  "neurodevelopmental disorders"  OR  "pervasive developmental disorder*") AND  TITLE-ABS-KEY ( mindfulness  OR  "mindfulness-based therap*"  OR  "mindfulness-based intervention*"  OR  yoga  OR  "mind-body therap*"  OR  meditation )  AND  TITLE-ABS-KEY ( "social behavior"  OR  "social behaviors"  OR  "emotional regulation" OR “emotional dysregulation” OR  well-being  OR  wellness  OR  "mental health"  OR  anxiety  OR  depression  OR  aggress*  OR  microaggression*  OR  attention  OR  stress  OR  "quality of life"  OR  communication  OR  health )  AND  TITLE-ABS-KEY ( child*  OR  youth  OR  "young people"  OR  "young peoples"  OR  "emerging adult"  OR  "emerging adulthood"  OR  "emerging adults"  OR  adolescent*  OR  "young adult"  OR  "young adulthood"  OR  "young adults" ) )  AND  ( LIMIT-TO ( LANGUAGE ,  "English" ) )
