# Supplementary material for: The efficacy of mindfulness-based therapy for anxiety, social skills, and aggressive behaviors in children and young people with Autism Spectrum Disorder: A systematic review
Source: Front Psychiatry. 2023 Mar 13;14:1079471. doi: 10.3389/fpsyt.2023.1079471 (PMC10040538; doi:10.3389/fpsyt.2023.1079471)
Supplement: Supplementary file 2 [file Table_1.docx]

# Appendix B: Characteristics of Studies Included in Systematic Review: Expanded

**Table 1**

*Characteristics of Studies Included in Systematic Review - Expanded*

| Citation | Design | Participants | | | | Intervention | Outcome measures | Country, Context and Instructor(s) | Relevant Findings |
| --- | --- | --- | --- | --- | --- | --- | --- | --- | --- |
|  |  | *Demographics* | | | *Control/*  *Comparison* |  |  |  |  |
|  |  | Total *n*  (% male) | *M* Age years *(SD);* Range years | Diagnosis  (Measures) |  |  |  |  |  |
| Ahemaitijiang et al. (2020) | Multiple-Baseline Design | *n* = 3 (100%) | 15.3 (1.2); 14-17 | ASD  (DSM-5) | - | Meditation on the Soles of the Feet (SoF)  Participants trained for 3 wks, 6 h total The SoF training and practice phase was in effect for 32-37 wks | Mother collected observable data on verbal aggression, physical aggression, and destructive behavior | China, Home - Mothers taught intervention, then mother instructed child | Post-intervention, all three adolescents showed reduction in verbal aggression, physical aggression, and destructive behavior. All three adolescents displayed no physical aggression during follow-up. Weighted average Tau-U scores for all three categories showed a strong treatment effect |
| Black & Rosenthal (2015) | Case Studies/  Interview | *n* = 6 (83.3%) | 18.8 (3.4); 16-24 | ASD  (DSM-IV-TR) | - | Transcendental meditation (TM)  15-20 minutes twice a day, minimum 10 times a week for at least 3 months | Semi-structured clinical interview with participants and parents - open and close ended questions | Home - Self-instructed | Participants reported that TM improved emotion and behavior regulation, the ability to tolerate and cope in novel settings and social environments, and the capacity to transition and manage unexpected changes in routine |
| Chan et al. (2013) | Randomized Controlled Parallel Trial | *n* = 40  (90%) | 12.4 (3.3);  6-17 | ASD (ADOS-2) | *N* = 20  (85%)  ASD  (ADOS-2)  PMR Intervention | Nei Yang Gong Chinese Meditation 2x a week for 4 weeks | The Tower of London Test – Drexel Version (TOLDX)  The second trial of the Children’s Color Trails Test (CCTT-T2)  The Five Point Test (FPT)  Parent completed: Autism Treatment Evaluation Checklist  and additional questionnaire. EEG | China, Clinic - Two clinical psychologists,  10 years’ experience in  Nei Yang Gong and PMR respectively | The experimental group demonstrated significantly greater improvement in self-control than the control group, which concurs with the parental reports of reduced autistic symptoms and increased control of temper and behaviors |
| Conner et al. (2019) | Within subject pre and post comparison design | *n* = 17 (88.2%) | 14.9 (1.54);  12-17 | ASD  (ADOS-2) | - | Emotion Awareness and Skills Enhancement Program (EASE) Mindfulness-based intervention targeting emotional regulation for people with ASD  16 weekly, 45-50 minute sessions | ABC-I, CGI, EDI  PROMIS Anxiety and Depression Scales RSQ - Social Stress Treatment Satisfaction Scale Treatment Fidelity Scale | America, Clinic – 9 therapists, including psychologists, rehabilitation counsellors, doctoral students in psychology, and a research clinician | Medium to large effects were seen in reduction of emotional regulation impairments as well as problem behaviors |
| de Bruin et al. (2014) | Within-group pre- and post- comparison design | *n* = 23 (73.9%) | 15.8 (2.7); 11-23 | ASD  (*N* = 4) Asperger’s (*N* = 8)  PDD  (*N* = 11)  (DSM-IV-TR, ADOS-G) | - | Mymind: Mindfulness training for Youngsters with ASD 9 weekly 1.5 hr sessions | AQ  MAAS-A  PSWQ  RRS  WHO-5  SRS | Clinic – Mental health-care professionals with ASD experience | Significant improvement on social skills, specifically in communication, cognition, and preoccupations |
| Hartmann et al. (2019) | Single Subject Design | *n* = 7 (57.1%) | 21 (2.6);  18-24 | ASD | - | 90-minute sessions, once a week for 12 weeks mindfulness-based intervention including didactic portions identifying emotions and practicing emotion regulation and social interaction | SRS  ERQ  SPAI-23  BPAQ | Clinic – Co-facilitated by two senior clinicians, with three assistants | There were no group-level improvements in emotion regulation (BPAQ, ERQ, SPAI-23), although three participants improved on suppression and two participants improved on reappraisal in the ERQ. |
| Ho et al. (2021) | Randomized Control Trial | *n* = 37 (76%) | 13 (2.3);  10-18 | ASD  (DSM-5) | Waitlist Control, n = 18, ASD | Mymind: 90 minutes sessions, once a week for 9 weeks | SRS  CBCL  BRIEF | China, Clinic - Trained mindfulness psychologists or social workers | Both the MYmind group and the waitlist control group showed improvement in social responsiveness and internalizing problems |
| Hwang et al. (2015) | Pilot Case Study | *n* = 6 (83.3%) | 11.5 (2.2);  8-15 | ASD Asperger’s PDDNOS | - | Mothers completed an 8-week mindfulness training program with weekly 2.5-h sessions. Children were provided with parent-mediated home-based mindfulness training for a period of 12 months including weekly home visits and online meetings with authors. | Freiburg Mindfulness Inventory  Parenting Stress Scale  Family Quality of Life The Child Behavior Checklist | Home - First and second authors, mothers taught mindfulness to their children | Reduction in aggressive behaviors following mother’s mindfulness intervention, with further reduction following child’s mindfulness intervention |
| Joshi & Rathi (2019) | Randomized Control Trial | *n* = 30 | -  4-16 | ASD  (ISAA) | Control  *N* = 15  ASD  (ISAA) | Integrated Approach of Yoga Therapy, 1hr, five days a week for 12 weeks | ABC SCQ | India, Clinic - Yoga Therapist, assisted by 4 shadow teachers | ABC scores were decreased in the yoga group (5.06%) without significance, control group scores increased (0.27%) with significance (p=0.03) |
| Kemeny et al. (2021) | Randomized Crossover Design | *n* = 27 (74%) | 16.3 (2.77); 12-21 | ASD | Therapeutic Riding Protocol - Crossover Comparison | HeartMath (HM) mindfulness-based intervention 1 hr per week for 10 weeks = 10 sessions total | SRS | Clinic - HeartMath Intervention Specialist | In the HM phase, parents reported decreases in anti-social skills such as behaving strangely, difficulty with changes in routine, and wandering aimlessly between activities. |
| Pahnke et al. (2014) | Quasi-experimental two-group trial (intervention/waiting list) study with repeated measures | *n* = 28 (75%) | -  13-21 | ASD  (DSM-IV) | Waitlist control  *N* = 13 (92%) ASD  (DSM-IV) | ACT-based skills training group programme based on mindfulness and acceptance, along with daily mindfulness exercises (6-12 minutes daily for 6 wks in class) Group sessions - 2 x 40 mins x 6 weeks | Strengths and Difficulties Questionnaire  Beck Youth Inventories | Sweden, School - Graduate psychology student under supervision of a mindfulness instructor and ACT therapist. Mindfulness training was facilitated by the classroom teacher | Self-reported prosocial behavior increased |
| Radhakrishna (2010) | Within subject pre- and post- comparison design | *n* = 6  (83.3%) | -  8-14 | ASD  (DSM-IV-TR) | - | Yoga therapy five times a week 45 minutes daily for 10 months. | Questionnaire and observers’ comments and interviews completed by parents and educators, pre- mid- and post-intervention | Clinic - Yoga instructor and mother present | Improvement in children’s imitation skills. Parents reported change in the play pattern of these children with toys, peers, and objects at home. Increased skills in eye contact, sitting tolerance, non-verbal communication, and receptive skills. |
| Ridderinkhof et al. (2018) | Repeated Measures | *n* = 45 (80%) | 13 (2.72);  8-19 | ASD  (DSM-IV & ADOS-G) | - | MYMind Child Program (9 wk x 1.5 h) plus one booster session 9 weeks after final session (1.5 h) | SRS CBCL | Netherlands, Clinic - Child and family mental health care professionals | Social communication problems decreased, and their emotional and behavioral functioning improved. |
| Ridderinkhof et al. (2019) | Qualitative | *n* = 14 (57%) | 12.4 (2.4);  9-17 | ASD, Asperger’s, PPD-NOS | - | MYMind Child Program (9 wk x 1.5 h) plus one booster session 9 weeks after final session (1.5 h) | Self- and Parent Report | Netherlands, Clinic - Mental Health Professionals | Improvement in social skills as reported by parents, however not at a significant level |
| Salem-Guirgis et al. (2019) | Within-subjects repeated measures | *n* = 23 (82.6%) | 15.6 (2.6); 12-23 | ASD | - | MYMind Program for children and adolescents with ASD, and their parents 9 weekly sessions and one booster session, which occurred 9 weeks post-program (1.5 h each) | Behavior Assessment System for Children SRS | Canada, Clinic - Two clinical psychologists and three behavioral consultants. Two psychology post-doctoral fellows and 6 clinical psychology graduate students co-facilitated | Improvement in emotion regulation and adaptive skills. |
| Scroggins et al. (2016) | Case study | *n* = 1  (100%) | 7  - | Apert Syndrome & ASD | - | Multisensory Yoga Sessions 2 x 45 mins per week for 4 weeks | Sparks Target Behavior Checklist Treatment and Research Institute for ASD Social Skills  Field Notes | Clinic - Trained yoga instructor | Less disruptive behaviors, specifically in shyness and unusual mannerism of looking around. Improved self-regulation during stressful situations outside the yoga. |
| Sharma & Sharma (2016) | Between subjects design | *n* = 20  - | -  8-14 | ASD | Control | Yoga 6 days per week for 90 weeks, 2 x sessions per day for 2 hours | Observational report from parents and teacher | India, School | Reduction in self-injurious behavior and aggression |
| Singh, Lancioni, Singh et al. (2011) | Multiple-baseline design across subjects | *n* = 3  (100%) | 15.3 (2.05)  13-18 | Asperger Syndrome | - | Meditation on the Soles of the Feet 5 days x 15 minute daily sessions. Practice 2 x per day and when required. Technique practiced until 3 consecutive weeks of no aggressive behavior | Parent and sibling observational report | Home - The mother of each adolescent had been trained to use SoF by an experienced trainer a month prior to the beginning of baseline. | Reported improvements in aggressive behaviors, less physical aggression observed during intervention and at follow-up |
| Singh, Lancioni, Manikam et al. (2011) | Multiple-baseline design across subjects | *n* = 3  (100%) | 15.6 (1.24)  14-17 | ASD | - | Meditation on the Soles of the Feet. Daily 30 min sessions x 5 days. Practice 2 x per day and when required. Technique practiced until 4 consecutive weeks of no aggressive behaviors. | Parent and sibling observational report | Home - The mothers were taught SoF by the senior author and instructed to practice the procedure themselves for a month prior to teaching it to their child | Reported improvements in aggressive behaviors, less physical aggression observed during intervention and at follow-up |
| Singh et al. (2018) | Multiple-baseline design across subjects | *n* = 4  (100%) | 11.2 (0.82)  10-12 | ASD  (DSM-5) | - | SOBER Breathing Space (informal mindfulness practice) 3 x 1h sessions, during the first week of training, followed by 2 x 30 min sessions, per week for the next 3 weeks = 6h total | Parent observational report | Clinic - Experienced mindfulness trainer | Reported improvements in aggressive behaviors, less physical aggression observed during intervention and at follow-up |
| Singh et al. (2019) | Multiple-baseline design across subjects | *n* = 3  (100%) | 16.6 (0.47)  16-17 | ASD | - | Surfing the Urge (informal mindfulness practice). Training: 3 x 30 min sessions twice a week for 2 weeks. Intervention then used independently as needed | Parent and educator observational report | Clinic - Experienced mindfulness trainer | Both parents and educators reported a significant decrease in aggressive behaviors |
| Sotoodeh et al. (2017) | Randomized Control Trial | *n* = 29  (72.4%) | 11.2 (2.91)  7-15 | ASD  (DSM-5 &  ADI-R) | Control  *N* = 14  (72.4%) | 8 week Yoga Training Program 24 sessions x 30 mins each | ATEC-II | Iran, Clinic – Three expert trainers who were certified to work with ASD children | A significant increase in social skills, as measured by ATEC social skills subscale, was found |
| Tanksale et al. (2021) | Randomised Control Trial with mixed repeated measures | *n* = 61 (67.7%) | 9.4(1.3); 8-12 | ASD | Waitlist control *n* = 30 (60% male), ASD | Incredible Explorers group program that combines Yoga-informed Mind Body Practice with Cognitive Behavioral Therapy | BRIEF-2  ASC-ASD – Parent report and Self-report  GAS  EAQ | Australia, Clinic - Clinical Psychologist and psychology student volunteers | Significant changes in verbal communications of emotions, and willingness to understand emotions. No improvement in anxiety except for self-reported performance anxiety. |

*Note.* ABC and -I = Aberrant Behavior Checklist and Irritability Subscale, ASC = Anxiety Scale for Children, ATEC and -II = Autism Treatment Evaluation Checklist and Second Edition, BPAQ = Buss Perry Aggression Questionnaire, BRIEF-2 = Behavior Rating Inventory of Executive Function, CBCL = Child Behavior Checklist, EAQ = Emotional Awareness Questionnaire, SCQ = Social Communication Questionnaire, SDQ = Social Difficulties Questionnaire, SRS and SRS-2 = Social Responsiveness Scale and 2nd Edition, STBC = Sparks Target Behavior Checklist, TRIAD = Treatment and Research Institute for ASD Social Skills Assessment, YSR = Youth Self Report (CBCL)
